# High Molecular Weight ACTH-Precursor Presence in a Metastatic Pancreatic Neuroendocrine Tumor Causing Severe Ectopic Cushing's Syndrome: A Case Report

**DOI:** 10.3389/fendo.2020.00557

**Published:** 2020-08-13

**Authors:** Roopa Mehta, César Ernesto Lam-Chung, José Miguel Hinojosa-Amaya, Paola Roldán-Sarmiento, Maria Fernanda Guillen-Placencia, Gerladine Villanueva-Rodriguez, Oscar Alfredo Juarez-Leon, Jefsi Leon-Domínguez, Mariana Grajales-Gómez, Jose Luis Ventura-Gallegos, Andrés León-Suárez, Francisco J. Gómez-Pérez, Daniel Cuevas-Ramos

**Affiliations:** ^1^Neuroendocrinology Clinic, Department of Endocrinology and Metabolism, Instituto Nacional de Ciencias Médicas y Nutrición Salvador Zubirán, Mexico City, Mexico; ^2^Pituitary Clinic, Endocrinology Division, Department of Medicine, Hospital Universitario “Dr. José E. González” UANL. Francisco I. Monterrey, Monterrey, Mexico; ^3^Biochemistry Department, Instituto Nacional de Ciencias Médicas y Nutrición Salvador Zubiran, Mexico City, Mexico

**Keywords:** ACTH, ectopic, neuroendocrine tumor, cushing syndrome, case report

## Abstract

Ectopic ACTH-secretion causing Cushing's syndrome is unusual and its diagnosis is frequently challenging. The presence of high-molecular-weight precursors throughout pro-opiomelanocortin (POMC) translation by these tumors is often not reported. We present the case of a 49-year-old woman with a 3-month history of proximal muscular weakness, skin pigmentation, and weight loss. Upon initial evaluation, she had a full moon face, hirsutism, and a buffalo hump. Laboratory workup showed hyperglycemia, hypokalemia and metabolic alkalosis. ACTH, plasma cortisol, and urinary free cortisol levels were quite elevated. Serum cortisol levels were not suppressed on dexamethasone suppression testing. An octreo-SPECT scan showed enhanced nucleotide uptake in the liver and pancreas. Transendoscopic ultrasound-guided biopsy confirmed the diagnosis of a pancreatic ACTH-secreting neuroendocrine tumor (NET). Surgical excision of both pancreatic and liver lesions was carried out. Western blot analysis of the tumor and metastases revealed the presence of a high-molecular-weight precursor possibly POMC (at 30 kDa) but not ACTH (normally 4.5 kDa). ACTH-precursor secretion is more frequent in ectopic ACTH-secreting tumors compared with other causes of Cushing's syndrome. Hence, the measurement of such ACTH precursors warrants further evaluation, especially in the context of ACTH-dependent hypercortisolism.

## Introduction

Ectopic ACTH syndrome (EAS) is the cause of 5–10% of ACTH (adrenocorticotropic hormone) dependent Cushing's syndrome (1, 2). Ectopic ACTH syndrome can be a paraneoplastic manifestation of several kinds of neuroendocrine tumors, most commonly bronchial (well-differentiated carcinoid or poorly differentiated small cell), thymic, or pancreatic (3) but also occasionally pheochromocytoma, medullary thyroid carcinoma (MTC), and prostate carcinoma (3). Neuroendocrine tumors (NET) are heterogeneous in nature and can originate in the gastrointestinal tract, bronchi, thyroid and pancreas. The clinical presentation can range from neoplasms with indolent growth to rapidly advancing hormone-secreting carcinomas, and from occult to large symptomatic tumors (4). Early diagnosis and localization of the ectopic source of ACTH is crucial, in order to permit the complete excision of the tumor, avoiding adrenalectomy and reducing the risk of metastatic disease. However, in up to 50% of the cases, the source of ACTH secretion cannot be found despite imaging studies (5).

The diagnostic approach to ACTH dependent Cushing's syndrome (CS) relies on evaluating the clinical presentation, biochemical tests (including dynamic testing with dexamethasone), and inferior petrosal sinus sampling (IPSS). Inferior petrosal sinus sampling is the gold standard test for distinguishing between pituitary and ectopic ACTH-secreting tumors, this invasive procedure is not widely available and shows a specificity of only 67%, with false-positive and –negative results (6). The next step is the localization of EAS, this relies on conventional imaging techniques, including computed tomography (CT) and/or magnetic resonance imaging (MRI). Since ACTH-producing tumors express somatostatin receptor, somatostatin analogs (SSA) scintigraphy or gallium-68-somatostatin receptor PET/CT may also be helpful in localizing the tumor.

Additional tools, for the diagnosis of EAS, include the possibility of utilizing tumor specific differences in peptide processing. Some useful biomarkers, include pro-opiomelanocortin (POMC) and agouti-related protein (AgRP), especially in clinically challenging cases (7). AgRP is an hypothalamic neuropeptide that modulate energy balance, essential for energy homeostasis (8). Pro-opiomelanocortin is a complex precursor that includes various peptide hormones such as ACTH, melanocyte-stimulating hormone (MSH) alfa, and beta-endorphin (9). Pro-opiomelanocortin is a 241 amino acid protein with a molecular weight of 28–30 kDa. In EAS, there is often incomplete processing of POMC by the tumor, resulting in high serum levels of ACTH precursors (7).

In this report we describe the case of severe Cushing's syndrome caused by a metastatic pancreatic neuroendocrine tumor associated with the presence of high molecular weight ACTH-precursor molecules, possibly POMC, detected in the tumoral tissues.

## Case Presentation

**Figure 1:**
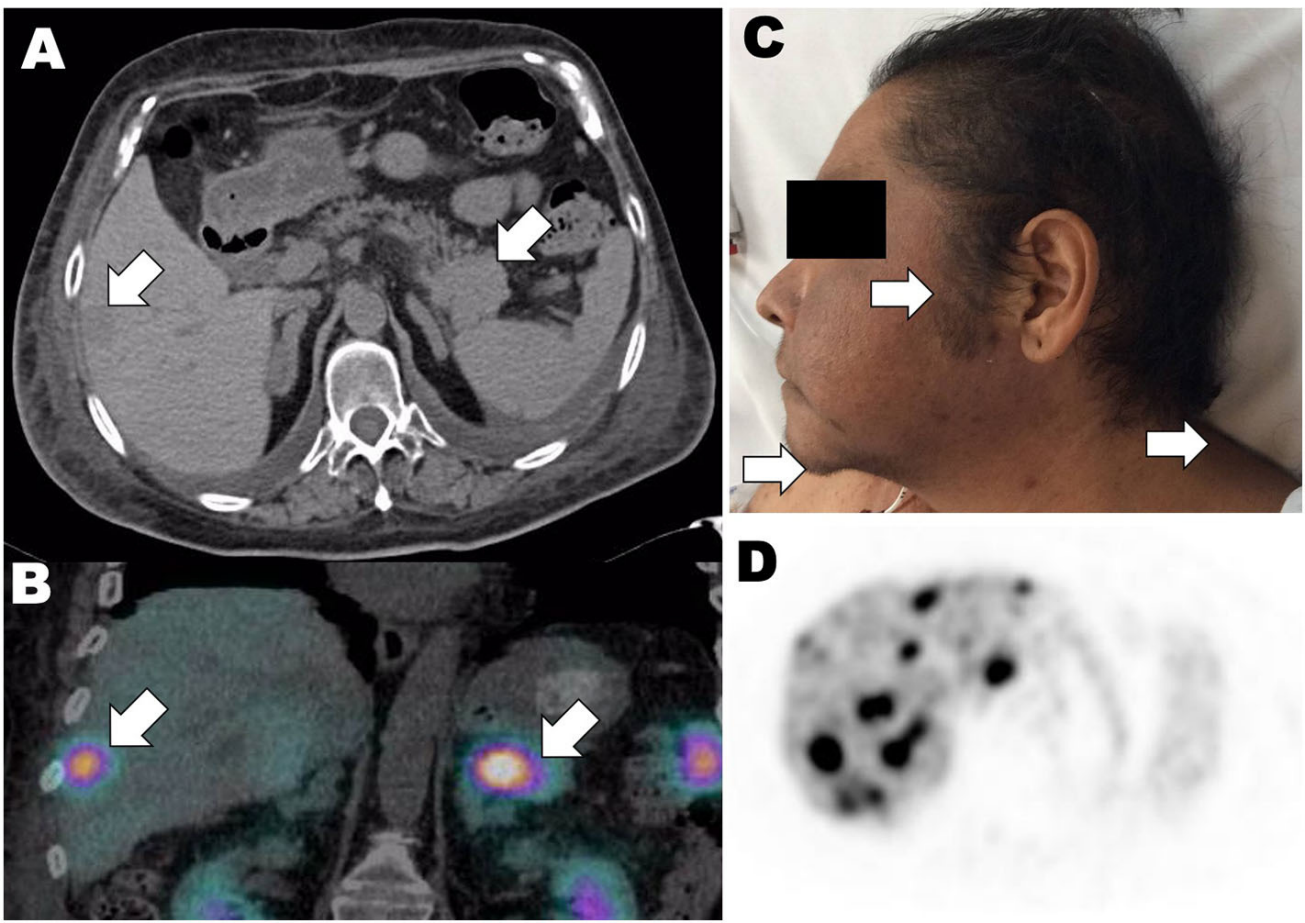
**(A)** Computerized tomography showing neuroendocrine tumor at the pancreatic tail causing liver metastases (white arrows). **(B)** Octreotide-single-photon emission computerized tomography (SPECT) study showing hepatic and pancreatic focal lesions with enhanced uptake (white arrows). **(C)** Clinical picture of the patient remarking the skin hirsutism (white arrows), and hyperpigmentation. **(D)** 18-Fluorodeoxyglucose (18-FDG) positron emission tomography-computerized tomography (PET-CT) 18 months after diagnosis with persistence of tumor liver metastases (black spots).

A 49-year-old Mexican, post-menopausal woman, of low socioeconomic status, was admitted to our institution with a 3-month history of proximal muscular weakness, skin hyperpigmentation, and bilateral ulcers on the lower limbs. She also mentioned unexplained weight loss (at least 6 kg) over the past 6 months. She had a past medical history of epilepsy, and a recent diagnosis of diabetes mellitus managed with metformin. She was not under further treatments or hormone replacement therapy. Physical examination revealed an overweight patient with a rounded “full moon” face, hirsutism (Figure 1C), mucocutaneous hyperpigmentation, a buffalo hump, upper extremity bruising, generalized weakness, and muscle wasting evident in the lower limbs. No skin striae where present. Thyroid examination was normal. Further clinical information is summarized in Table 1. The patient had significantly high ACTH [1,070 pg/mL (10–60)], plasma cortisol am [41.6 ug/dL (6–22.6)], and 24 h urinary cortisol levels [9,263 ug/24 h (<140), Table 2]. Other laboratory studies showed hyperglycemia [490 mg/dL (70–99)], low potassium levels [2.4 mmol/L (3.5–5.1)], and a metabolic alkalosis [bicarbonate 46.7 mmol/L (10–14), Table 1].

**Table 1:** Clinical and laboratory data at diagnosis.

**Parameter**	**Result**	**Reference ranges**
**Clinical data**		
Age	49 years	–
Weight	64 kg	–
BMI	26.3	–
Blood pressure	100/60	–
Heart rate	128	–
Respiratory rate	28	–
Temperature	36.7°C	–
**Laboratory data**		
Albumin	3.2	3.5–5.7 mg/dL
pH	7.51	7.31–7.41
HCO3	46.7	24–28 mmol/L
PCO2	59	28–40 mmHg
HbA1C	14	<5.7%
DHEA-S	41	35–430 ug/dL
DHEA	3.5	0.2–9.8 ng/mL
Free testosterone	62.3	0–2.03 pg/mL
Total testosterone	3.6	0.1–0.75 pg/mL
Glucagon	127	59–271 pg/mL
Chromogranin A	5.7	<3 nmol/L
Gastrin	304	13–115 pg/mL
VIP	11	0–30 pmol/L
CRH	2.1	<10 pg/mL

*ACTH, adrenocorticotropin; DHEA, dehydroepiandrosterone and DHEA-S, sulfate; VIP, vasoactive intestinal peptide; CRH, corticotropin releasing hormone*.

**Table 2:** Baseline cortisol and ACTH values and results of continuous 7 h IV infusion of 7 mg dexamethasone suppression test.

	**On admission**	**Reference range**
Cortisol a.m.	41.6 ug/dL	6–22.6 ug/Dl
Cortisol p.m.	60 ug/dL	6–22.6 ug/dL
24 h-urinary free cortisol	9262.88 ug/24 h	<140 ug/24 h
ACTH	1,070 pg/mL	10–60 pg/mL
**7 mg dexamethasone infusion test (during 7 h)**		
*Time before dexamethasone infusion started*		
Cortisol a.m.30 min (7:30 AM)	41.66 ug/mL	Average: 41 ug/dl Normal <22
Cortisol a.m.15 min (7:45 AM)	41.18 ug/mL	
Cortisol a.m.0 min (8:00 AM)	40.23 ug/dL	
*Time before infusion of dexamethasone ended*		
Cortisol p.m.30 min (14:45 h)	38.18 ug/dL	Average: 39 ug/dl Normal <10
Cortisol p.m.15 min (15:00 h)	40.53 ug/dL	
Cortisol p.m.0 min (15:15 h)	38.94 ug/dL	
*Next morning after dexamethasone infusion test*		
Cortisol a.m.0 min (8:00 AM)	42.01 ug/dL	Average: 41 ug/dl Normal <10
Cortisol a.m.15 min (8:15 AM)	41.18 ug/dL	
Cortisol a.m.30 min (8:30 AM)	40.68 ug/dL	
**Laboratory hormone results after surgery**		
Plasma cortisol AM	14.34	6–22.6 ug/dL
Plasma cortisol PM	9.52	6–22.6 ug/dL
24 h-urinary cortisol	111.7 ug	<140 ug/day
ACTH	24	10–60 pg/mL
DHEA	0.51	0.2–9.8 ng/mL
Total testosterone	0.17	0.1–0.75 pg/mL

*ACTH, adrenocorticotropin; DHEA, dehydroepiandrosterone*.

**Figure 2:**
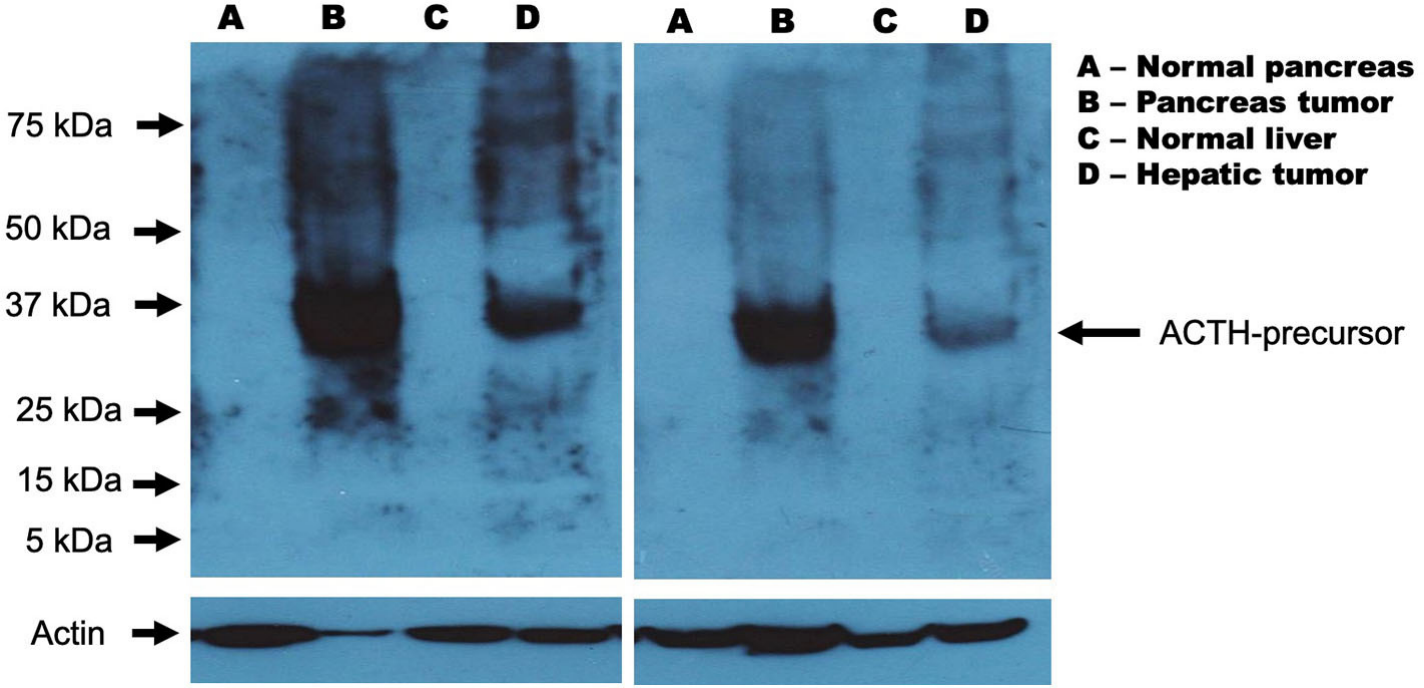
Western blot analysis from samples of pancreatic tumor and liver metastasis. **(A)** Normal pancreas; **(B)** Pancreatic NET; **(C)** Normal liver; **(D)** Liver metastasis. Pancreatic and liver tissues were homogenized in the presence of RIPA buffer (PBS with detergents and protease inhibitors cocktail). Total protein concentration was determined using the commercial Bradford reagent assay (Bio-Rad, Hercules, CA). Whole protein (200 μg) was used for the detection of the ACTH protein. Samples were first boiled in sample buffer (125 mM Tris-HCl, pH 6.8, 1% v/w SDS, 10% v/v glycerol, 0.1% bromophenol blue, 2% v/v 2 alfa-mercaptoethanol) for 5 min and separated by 12% SDS-PAGE. Then, the gels were transferred to PVDF membranes (Merck Millipore Corporation, Darmstadt Germany) using a Trans-Blot Cell system (Bio-Rad, Hercules, CA) in transfer buffer (25 mM Tris, 190 mM glycine, and 10% methanol) at 40 V overnight. The following day, the membranes were blocked with non-fat dried milk (5%) dissolved in TBS buffer (150 mM NaCl, 20 mM Tris, and 0.1% Tween) and probed overnight at 4 °C with rabbit monoclonal anti-ACTH antibody (Abcam, Cambridge UK) diluted 1:2,000 in TBS buffer with BSA (150 mM NaCl, 20 mM Tris, 0.1% Tween, and 1% BSA at pH 7.5). After washing, the membranes were incubated for 1 h with anti-rabbit immunoglobulin-HRP (Thermo Scientific, Rockford, IL USA). The signals were detected by enhanced chemiluminescence using the SuperSignal^TM^ system (Thermo Scientific, Rockford, IL USA) on X-ray film (Kodak, USA). As the control loaded, actin was simultaneously detected, using mouse anti-human β-actin antibody (Santa Cruz Biotechnology, Santa Cruz CA, USA). The signal was developed using anti-mouse immunoglobulin-HRP (Thermo Scientific, Rockford, IL) and chemiluminescence system.

This prompted a workup to investigate the possibility of ACTH-dependent CS. A high-dose dexamethasone suppression test with a continuous 7 h IV infusion of 7 mg dexamethasone (1 mg/h, as described by Biemond and Bogaert) was carried out (15). Absence of cortisol suppression at morning of day 2 confirmed the presence of Cushing's syndrome, and, the lack of cortisol suppression at afternoon of day 1, clearly suggested an extra-pituitary source of hypercortisolism with a very low probability of a pituitary corticotroph adenoma (Table 2). Ectopic ACTH-secretion syndrome was therefore suspected. Diagnostic approach was completed with serum chromogranin A, total and free testosterone levels, all found elevated, while gastrin, VIP, CRH, dehydroepiandrosterone (DHEA), and DHEA-sulfate (DHEA-S) levels resulted on normal range (Table 1). Because rapid and aggressive onset of symptoms, the severity of the hypercortisolism (both clinically and biochemically), the high levels of ACTH, and the lack of suppression of cortisol at afternoon of day 1 during dexamethasone 7 mg infusion test (Table 2), the IPSS was not considered necessary in this case, as pituitary corticotroph adenoma was very unlikely. Instead, a computerized tomography study was performed, and result showed a 2.7 cm hypodense hepatic nodule, with a 4.3 cm distal pancreatic lesion, and bilateral diffuse adrenal hyperplasia (Figure 1A). The metabolic activity of these lesions was investigated using an octreotide-single-photon emission computerized tomography (SPECT) study. This showed increased metabolic activity in both tumors (Figure 1B). We considered the possibility of ectopic-ACTH secreting tumors at these sites. Therefore, the pancreatic lesion was biopsied using trans-endoscopic ultrasound (TEUS) and a well-differentiated pancreatic neuroendocrine tumor was confirmed. Surgery excision of both tumors was programmed. Preparation involved the use of ketoconazole, titrated up to 1,200 mg QD, in order to control cortisol levels before surgical excision. Distal pancreatectomy and liver metastasis resection were carried out without any acute complications. The pathology report established the diagnosis of a grade 2 well-differentiated neuroendocrine pancreatic neoplasm positive for chromogranin A, synaptophysin, and Ki-67 (15%) (16). In order to confirmed ACTH synthesis from both pancreas and liver tumors, we performed western blot (WB) analysis for ACTH, on both human histological samples obtained from the patient. Results showed a 30 kDa protein in both pancreatic and liver tumors, but negative on normal pancreas and liver as control (Figure 2). Also, WB did not show any protein at ACTH molecular weight of 4.5 kDa. Taking all together, WB results suggested the presence of a high molecular weight ACTH-precursor such as POMC (Figure 2).

Ketoconazole was gradually withdrawn after surgery, and corticosteroid replacement therapy with hydrocortisone was provided for 3 days after the surgical intervention. Morning and afternoon cortisol levels, as well as 24-hour urinary free cortisol were measured and were reported in the normal range (Table 2). Unfortunately, postoperative hospitalization was complicated by a pancreatic fistula, pulmonary embolism, and hospital acquired pneumonia. Conservative management for the pancreatic fistula was undertaken. The pulmonary embolism and pneumonia were appropriately treated and resolved. The patient was discharged with an outpatient visit scheduled within a month.

**Figure 3:**
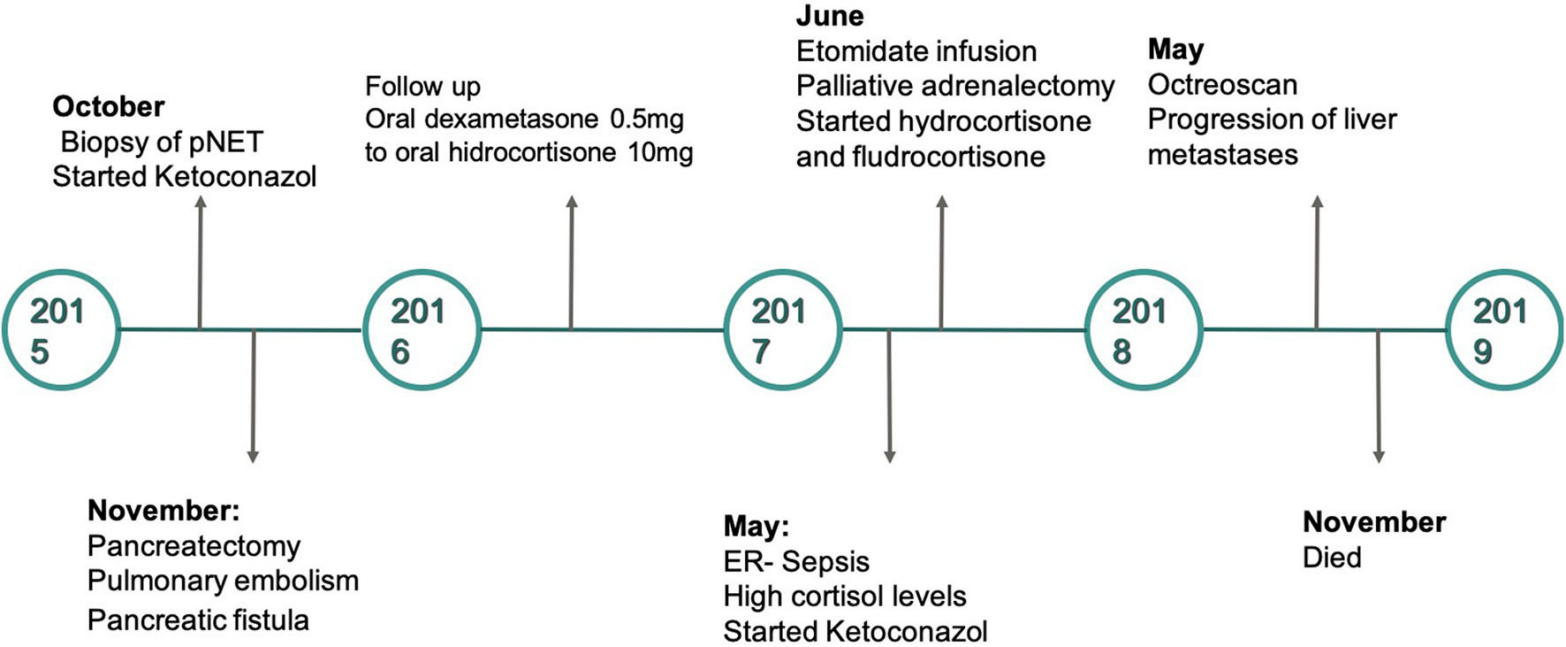
Timeline of the case presented.

Following 18 months of ambulatory follow-up, the patient was admitted to the emergency room with diffuse abdominal pain, vomiting, and muscle weakness. High ACTH (1,000 pg/ml), morning cortisol levels (62 ug/dl), UFC (3,509 ug/24 h), together with hypokalaemia (3.08 mmol/L) were recorded. An urgent bilateral adrenalectomy was programmed to definitively control the hypercortisolism. In preparation for the surgery, an etomidate infusion was started (initial dose 0.04 mg/kg/h) and titrated to control cortisol levels (using daily 24-h urinary cortisol and plasma cortisol levels). The dose of the drug was then gradually decreased. The day before surgery, the infusion was stopped; and 8 h before surgery, hydrocortisone replacement therapy was started using 100 mg IV every 8 h. The adrenalectomy was carried out with no complications. Following the surgical procedure, prednisone with fludrocortisone replacement were started. The clinical picture of patient improved; however, 18-fluorodeoxyglucose (18-FDG) positron emission tomography-computerized tomography (PET-CT) confirmed the presence of inoperable liver metastases (Figure 1D). No further surgical intervention could be offered, and the patient eventually died. Her survival following the initial diagnosis was no >36 months (Figure 3). Written informed consent was obtained from the patient for this report. Nevertheless, her clinical perspective was not obtained.

## Discussion

Ectopic ACTH syndrome (EAS) represents one of the major diagnostic challenges in clinical endocrinology (17–19), regardless of the availability of broad diagnostic tests. In nearly half of the cases, the source of ectopic ACTH-secretion is situated in the lung, bronchial carcinoid tumors being the most common cause (20, 21). ACTH-secreting pancreatic neuroendocrine tumors are rare and more than 95% metastasize (22). As in the case of our patient, such neuroendocrine tumors usually present with liver metastasis since neoplastic cells from the pancreas enter the enterohepatic circulation (23) and plasma chromogranin A (CgA) is usually elevated (24).

An effort has been made to describe the clinical and pathologic features of ACTH-secreting pancreatic neuroendocrine tumors despite its rarity (25). Maragliano reviewed 134 cases (124 previously reported and 10 from their series), reporting a female preponderance (66%), occurring at a mean age of 42.1 years. This age average is almost 20 years less than the mean age compared to the overall pancreatic neuroendocrine tumors (26). More than a third of the cases, clinical presentation is accompanied with Zollinger-Ellison syndrome whereas only 5% presents with insulinoma, and 1% with carcinoid syndrome (25). Although serum CgA has been widely used as a diagnostic biomarker in pancreatic neuroendocrine tumors, it has a limited role, especially with localized diseases (27). However, serum alfa-feto protein elevation should prompt consideration of the presence of pancreatic acinar cell carcinoma and/or pancreatoblastomas since it is associated with worst prognosis (25). Radiolabeled somatostatin analogs diagnostic imaging such as PET/CT with gallium-68 DOTATATE is used for most patients in which the uptake of radiolabeled somatostatin analogs can anticipate the clinical response to therapy (28).

It has been suggested that the signs and symptoms of EAS are influenced by the tissue and type of tumor secreting ACTH (10). Pancreatic neuroendocrine neoplasms associated with EAS generally show a short time between the onset of clinical symptoms of hypercortisolism and diagnosis (6, 17). Our patient was diagnosed promptly, and rapidly underwent surgical treatment to control hypercortisolism. Aggressive EAS, can be associated with high POMC/ACTH ratio since the tumor tissue may secrete precursors that are not detected in conventional ACTH assays (11, 12). However, we were not able to evaluate POMC serum levels. First, we do not perform this evaluation as routine, and therefore, we did not have the assay ready throughout the diagnostic approach, and secondly, aggressiveness of the Cushing's syndrome required promptly medical and surgical therapy, and serum samples were not preserved before treatment was started. Instead, western blot was carried out as an effort to evaluate the presence of ACTH precursor molecules on this case. Figure 2 shows strong positiveness of a 30-kDa protein isolated from the pancreatic and hepatic tumor tissues, but not at their normal controls. ACTH is a 39 amino acid peptide with a 4.5 kDa molecular mass, synthesized from pre-pro-opiomelanocortin (pre-POMC). During translation, the signal peptide is removed and produces a 241-amino acid polypeptide POMC. After multiple post-translational modifications, POMC is proteolytically cleaved from amino acid 135–174 to synthesize ACTH (13). Using an immunoradiometric assay for the quantitative determination of whole corticotropic hormone (ACTH) in human plasma (ELSA-ACTH Cisbio Bioassays, Model 22), two monoclonal antibodies are prepared against sterically remote antigenic sites on the ACTH molecule. Detected serum ACTH levels in our patient, however, may in fact reflect quantification of same antigenic sites but from amino acid 135–174 at the ACTH-precursor POMC. Otherwise, it would be very difficult to explain the absence of ACTH on western blot from both tumors (pancreas and liver) in the context of a patient with an aggressive ACTH-ectopic secretion Cushing syndrome. In fact, cross-reactivity in ACTH assay with ACTH precursors have been reported up to 10% (14, 29, 30). We, therefore, can assess the possibility that POMC together with other intermediary proteins throughout POMC translation such as melanocyte-stimulating hormone alfa (MSH-alfa), contributed to the aggressive clinical presentation in our patient. Furthermore, the clinical utility of serum POMC levels have been demonstrated in differentiated Cushing disease and occult EAS, particularly when there is no detectable tumor or there is a pituitary microadenoma on MRI and IPSS is required (7, 31–34). Moreover, increased serum levels of AgRP can also be considered as a neuroendocrine tumor marker for EAS (7). Ectopic ACTH secretion associated with underlying malignancy is treated with surgery. Prognosis depends on the histological findings and the presence of metastasis. Also, regardless of primary lesion resection, and control of secreted hormone levels and clinical manifestations, recurrence rate is high, particularly, with liver metastases as in the case of our patient (35).

Non-surgical liver-directed therapies include ablation and hepatic arterial embolization. Ablation are mainly employed for liver metastasis or as an adjunct for surgical treatment or resection whereas hepatic arterial embolization, as a palliative care for symptomatic, non-resectable hepatic disease or an alternative for medical therapy (36). Cytotoxic chemotherapy, including capecitabine and temozolomide, are recommended in symptomatic patients from tumor mass or in the presence of a fast-growing metastasis (36). Therapeutic targets for neuroendocrine tumors include phosphatidylinositol-3-kinase (PI3K)/Akt and rapamycin (mTOR), both of which are involved in cell-cycle and growth control pathways in cancer (37, 38). Also, everolimus, an oral inhibitor of the mTOR pathway, and sunitinib, an oral multi-targeted tyrosine kinases inhibitor have been shown to improve progression-free survival in metastatic entero-pancreatic neuroendocrine tumors (39, 40). Lanreotide and octreotide, which are somatostatin analogs that act by binding to somatostatin type 2 receptors, are used to control symptoms. In addition, lanreotide prolongs progression-free survival in pancreatic NETs (41). Recently, peptide receptor-targeted radiotherapy (PRRT) in the treatment of metastatic NETs with a homogenous expression of SSTRs has been tested, mainly using ^90^Yttrium- DOTA-TOC or ^177^Lutetium- DOTA-TATE (42, 43). Response rates of 15–40% have been reported, although prospective randomized clinical trials comparing these agents with conventional treatment are lacking. Although the majority of NETs growth slowly with a relatively indolent course, up to 40% may show metastases at the time of diagnosis. Even in the presence of liver metastases, such as the case presented here, the majority of such patients may survive for many years with the use of these emerging therapies.

Although prognosis are variable, there are several factors that impact survival, including age, type of NET, histologic grade, hypercortisolism and metastases (3, 17, 44). In a series of NETs and EAS, pancreatic NETs had a lower curative surgical rate and a lower 5-year overall survival when compared to bronchial carcinoid EAS (45). The median survival is 50 months in patients with grade one or two pancreatic NETs and distant metastases (44).

To conclude, although we could not measure serum level of POMC, we were able to demonstrate its presence in two different tumor tissues (liver and pancreas) from a patient with an aggressive Cushing's syndrome. This case emphasizes the relationship between ectopic ACTH-secreting Cushing's syndrome and importance of ACTH precursors quantification such as POMC. The measurement of such precursors warrants further evaluation, especially in the workup of such cases.

## Data Availability Statement

The raw data supporting the conclusions of this article will be made available by the authors, without undue reservation.

## Ethics Statement

Written informed consent has been obtained from the patient for publication of the submitted article and images.

## Author Contributions

RM, CL-C, PR-S, GV-R, OJ-L, JL-D, MG-G, AL-S, and DC-R: diagnostic approach of the case, sample collection, writing the paper, editing images, and literature review. MG-P: diagnostic approach of the case, writing the paper, editing images, and literature review. JH-A: writing the paper, editing images, and literature review. JV-G: sample collection, preparation, and test. FG-P: diagnostic approach of the case, editing images, and literature review. All authors: read and approved the final manuscript.

## Conflict of Interest

The authors declare that the research was conducted in the absence of any commercial or financial relationships that could be construed as a potential conflict of interest.
